# The Efficacy of Andexanet Alfa for the Reversal of Factor Xa Inhibitors Is Not Influenced by Hemodilution with Different Volume Expanders

**DOI:** 10.3390/jcm13226706

**Published:** 2024-11-08

**Authors:** Jan Wienhold, Rolf Rossaint, Eline Vandeput, Oliver Grottke

**Affiliations:** Department of Anesthesiology, University Hospital RWTH Aachen, 52074 Aachen, Germany

**Keywords:** hemodilution, FXa inhibitors, hemorrhage, blood coagulation

## Abstract

**Background:** Andexanet alfa is a specific antidote for factor Xa (FXa) inhibitors. It is licensed to treat patients under FXa inhibitor therapy with life-threatening bleeding. Concomitantly, volume expanders are used to compensate for blood loss and maintain circulation. The competitive binding of andexanet to FXa inhibitors may be disrupted due to hemodilution, as shown by laboratory assays with high sample dilution. This study investigated the efficacy of andexanet for the reversal of FXa inhibitors under hemodilution. **Methods**: Blood from 10 healthy volunteers was anticoagulated with rivaroxaban and subsequently treated with four different volume expanders (Ringer’s solution, 4% gelatine, 5% and 20% human albumin (HA)) at two dilution levels (20% and 50%). After anticoagulation and hemodilution, andexanet was added according to the high-dose protocol. Blood samples were analyzed using a Russell’s viper venom (RVV) test on a Clot Pro^®^ device, a thrombin generation assay, a fully automated coagulation analyzer and a chromogenic anti-FXa activity assay. **Results**: After anticoagulation, the median rivaroxaban concentration was 272 ng/mL (IQR 254–353). Anticoagulation with rivaroxaban caused a significant impairment of all coagulation parameters, which was further aggravated by hemodilution. After the administration of andexanet, coagulation parameters in anticoagulated samples were reversed to near baseline in all groups. Andexanet administration decreased the rivaroxaban plasma concentration in all groups to a median of <10 ng/mL. In the anticoagulated, non-hemodiluted samples, anti-FXa activity was reduced by 98%. The anti-FXa activity in the anticoagulated, hemodiluted samples was reduced by approximately 96% in the 20% diluted samples and by about 93% in the 50% diluted samples. **Conclusions**: Our data indicate that FXa inhibitor reversal with andexanet is about 5% less effective with 50% hemodilution than in non-hemodiluted samples.

## 1. Introduction

Direct oral anticoagulants (DOACs) have become increasingly important in clinical practice [1]. They have been shown to be superior to vitamin K antagonists (VKAs) in treating and preventing thrombotic events while carrying a reduced risk of bleeding [2,3]. Due to their mechanism of action, DOACs can cause or exacerbate coagulopathy in traumatized or severely bleeding patients [4]. Elevated plasma concentrations of DOACs and high-risk surgery are associated with a higher risk of major bleeding and higher mortality [5]. Prompt reversal of the anticoagulant effects of DOACs in critically bleeding patients is therefore imperative to mitigate the risk of exsanguination and minimize the need for transfusion.

There are specific and nonspecific approaches to restore hemostatic capacities in DOAC-anticoagulated patients. As a nonspecific measure, prothrombin complex concentrates can be used to restore hemostasis in patients on DOACs [6]. In FXa inhibitor-related life-threatening bleeding, the recombinant modified human FXa protein andexanet alfa (andexanet) was approved in 2019 by the European Medicines Agency (EMA) [7] as a specific reversal agent for rivaroxaban and apixaban. Andexanet is a protein without enzymatic activity, unable to cleave and activate prothrombin since the amino acid serine in the active binding site has been replaced by alanine. In addition, the γ-glutamyl carboxylase domain has been removed, impeding the formation of the prothrombinase complex. Clinical trials investigated the effectiveness and safety of andexanet, collectively referred to as the ANNEXA trials. The ANNEXA-A and -R trials showed a significant reduction in anti-factor Xa activity in a cohort of healthy subjects. In addition, increased average thrombin generation and D-dimers were observed after andexanet administration [8]. The ANNEXA-4 trial investigated andexanet in a single-group cohort study with major bleeding in patients on FXa inhibitors, such as apixaban and rivaroxaban, as well as a subset of patients on edoxaban and enoxaparin [9]. Anti-FXa activity was reduced by 92 to 95%, and despite a rebound within four hours, hemostasis was rated as “excellent” or “good” in 82% of the patients after 12 h. Thromboembolic events occurred in 10% of the patients within the 30-day follow-up period.

The recently published ANNEXA-I study investigated FXa inhibitor reversal with andexanet in patients with intracerebral hemorrhage [10]. Hemostatic efficacy was achieved in 67% of patients receiving andexanet and 53% of patients receiving usual care (mostly PCC). Anti-FXa activity in the andexanet-treated group could be reduced by 94% compared to 27% with PCC. The thromboembolic risk of 10% in patients treated with andexanet was consistent with the findings of the ANNEXA-4 trial.

The European guideline on the management of major bleeding and coagulopathy following trauma [11] recommends andexanet as first-line therapy to specifically reverse the effects of apixaban and rivaroxaban in life-threatening bleeding. Concomitantly with nonspecific hemostatic agents or andexanet, volume expanders are administered in bleeding patients to maintain blood pressure and tissue oxygenation [4,12]. Isotonic crystalloids as well as colloids can be used in the initial phase of treatment in bleeding trauma patients [4]. However, colloids have been shown to impair coagulation and should be restricted [11,13].

In cases of major bleeding associated with FXa inhibitor therapy, andexanet might be administered simultaneously with volume expanders to reverse the inhibitory effects of the FXa inhibitor. It is unknown how different volume expanders influence the effectiveness of andexanet in binding FXa inhibitors. This question becomes particularly relevant in light of the observation that andexanet has been shown to dissociate from FXa inhibitors under higher sample dilution due to its competitive binding [14]. A drug warning was issued in 2020 to use commercially available anti-FXa assays to monitor FXa inhibitor levels after reversal with andexanet [15]. Due to high sample dilution in the assay protocols, the andexanet–FXa inhibitor complex shifts from the bound to the unbound state. It remains unclear if this laboratory finding can be reproduced in a scenario involving massively bleeding patients being treated with volume expanders.

This study investigated the effectiveness of andexanet for reversing FXa inhibitors under hemodilution with four different volume expanders.

## 2. Materials and Methods

### 2.1. Study Design and Ethics

We conducted a single-center experimental in vitro study designed according to the extended CONSORT criteria [16]. The study was conducted at the Department of Anaesthesiology of the RWTH Aachen University Hospital. The study protocol complies with the Declaration of Helsinki on ethical principles for medical research involving human subjects and was approved by the local ethics committee on 1 July 2021 (EK215-21). Written informed consent was obtained from every donor.

### 2.2. Participants and Blood Drawing

Blood samples were drawn from ten healthy volunteers, five male and five female. The donors had no history of coagulation disorders and did not take any medications that might affect coagulation before venipuncture. Blood drawings were conducted with minimal stasis using trisodium citrate tubes (volume ratio of citrate/blood 1:9). The first milliliter after puncture was discarded. The whole blood samples were stored at room temperature before analysis and centrifuged within four hours after collection. Standard hematology parameters (hemoglobin, erythrocytes, white cell count and platelet count) were measured using a standard hematology analyzer (MEK-6108, Nihon Kohden, Rosbach, Germany).

### 2.3. Sample Preparation and Analytical Methods

**Figure 1:**
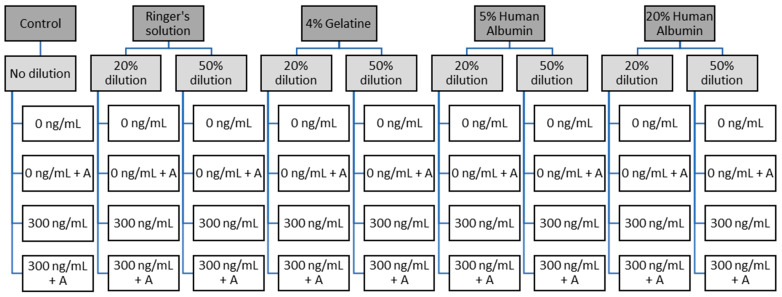
Overview of sample preparation. Values in ng/mL indicate rivaroxaban concentration. A = andexanat alfa added. A 50% dilution means that the whole blood aliquot was diluted with the same volume of the indicated volume expander.

Half of the blood volume was spiked with the FXa inhibitor rivaroxaban. The spiking dose was calculated to achieve a concentration of 300 ng/mL. After spiking, rivaroxaban concentration was controlled using a fully automated coagulation analyzer (ACL Top 550 CTS^®^, Werfen, Bedford, MA, USA). After baseline measurements, samples were diluted in vitro with either balanced Ringer’s solution (Sterofundin, B.Braun, Melsungen, Germany), 4% gelatine (Gelafundin, B.Braun, Melsungen, Germany) 5%, or 20% human albumin (HA; Biotest 5%, Biotest Pharma GmbH, Dreieich, Germany; Grifols^®^ 20%, Instituto Grifols, S.A., Barcelona, Spain) at two different dilution levels each (20% and 50%, Figure 1). Subsequently, andexanet was added at a concentration of 4 µM (equal to 164 µg/mL), corresponding to the peak plasma concentration observed in the high-dose protocol following an 800 mg bolus [17]. The andexanet dose was calculated based on the blood volume, excluding the added volume expander. Samples were incubated for five minutes on a roller mixer at room temperature.

#### 2.3.1. Viscoelastometry

Thirty-six whole blood samples were prepared (white boxes in Figure 1). These samples were analyzed using a Russell’s viper venom (RVV) test on a ClotPro^®^ device (Enicor GmbH, Munich, Germany). The parameters measured were mean clot firmness (MCF), clotting time (CT), and clot formation time (CFT).

The ClotPro^®^ is a relatively new viscoelastic testing device that can be used for point-of-care diagnostics. Whole blood samples are analyzed by rotational thromboelastometry, similar to other devices on the market (ROTEM^®^, TEG^®^). The ClotPro^®^ provides six independent channels with facilitated handling. The needed reagents are already stored in the tip of the pipette, making any handling with reagents unnecessary. Calcium chloride is automatically added to all tests for recalcification. The ClotPro^®^ device provides a new assay to detect FXa inhibitors directly, the RVV test. Russell’s viper venom activates FX and is, therefore, perfectly suited to detect the presence of FXa inhibitors as a point-of-care device in bleeding patients. A total of 340 μL of whole blood is pipetted electronically in a cup, and subsequently, a pin is inserted. The force required to rotate the cup relative to the pin is tracked. Once the coagulation is triggered and a clot forms, turning the cup becomes increasingly difficult. The force is recorded over time and translated into thromboelastic amplitudes [18]. After thromboelastic measurements, samples were centrifuged, and the cell-free plasma samples were frozen at −80 °C before further assessment.

#### 2.3.2. Plasmatic Coagulation Parameters and Thrombin Generation Assays

Platelet-poor plasma (PPP) samples were analyzed using a fully automated coagulation analyzer (ACL Top 550 CTS^®^, Werfen) according to the manufacturer’s instructions. Prothrombin time (PT; ReadiPlastin reagent; Werfen), activated partial thromboplastin time (aPTT; HemosIL^®^ SynthASil; Werfen) and fibrinogen concentration (HemosIL^®^ QFA Thrombin; Werfen) were assessed for all samples. Thrombin generation was measured in PPP samples using 5 pM tissue factor (TF) and 4 mM phospholipids (PL) with a Fluoroskan Ascent^®^ plate reader (Thermo Fisher Scientific OY, Vantaa, Finland). To determine lag time, endogenous thrombin potential (ETP) and peak thrombin formation, Thrombinoscope software (Thrombinoscope BV, version 4, Diagnostica Stago S.A.S, Asnières sur Seine Cedex, France) was used.

#### 2.3.3. Chromogenic Anti-FXa Assay

A modified Coamatic^®^ Heparin chromogenic assay (Diapharma, West Chester Township, OH, USA), calibrated for rivaroxaban, was used according to the manufacturer’s instructions to assess anti-FXa activity. The procedure in the presence of andexanet was identical to the previously described methods [19].

### 2.4. Statistical Analysis

This study was designed as an exploratory pilot trial. Therefore, no sample size calculation was performed. GraphPad Prism 10 (GraphPad Software, San Diego, CA, USA) was used for statistical analysis and figure creation. The Shapiro–Wilk test was performed to examine normal distribution. As not all data sets fulfilled criteria for normal distribution, the nonparametric Mann—Whitney U test was used to consistently assess statistical differences for all data sets. The significance level was set at α = 5%. Categorical and ordinal variables were summarized as count (percentage) and continuous variables as median [interquartile range].

## 3. Results

### 3.1. Blood Count

Blood samples were drawn from ten young, healthy volunteers: five female and five male. Standard hematology parameters were within the reference range (platelets (×10^3^/μL): 176 (159–197; hemoglobin (g/dL): 14 (13–15) and WBC (×10^3^/μL): 6 (4–7)). For the 50% dilution with balanced Ringer’s solution (R50), measurements could not be conducted in all samples due to enhanced clotting during whole blood measurements, hampering pipetting and thromboelastic measurements.

### 3.2. Plasma Rivaroxaban Levels

**Figure 2:**
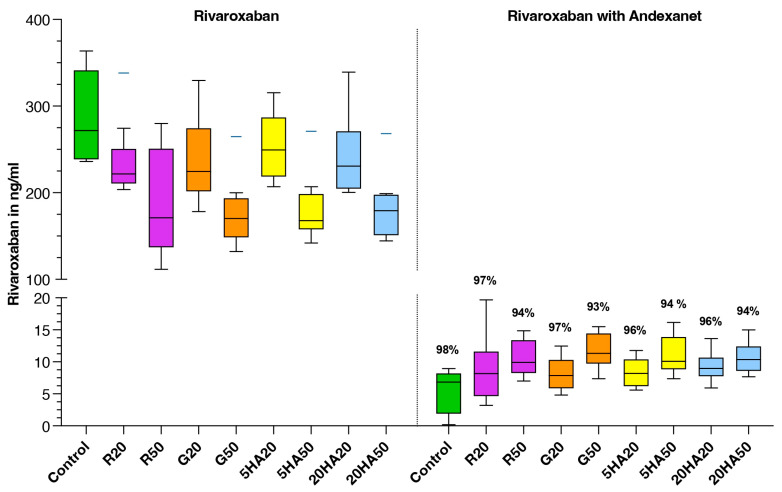
Rivaroxaban concentration in ng/mL measured by anti-FXa activity chromogenic assay. Data are shown as median values, boxes show IQR and whiskers extend to the minimum and maximum values. The percentages above the boxes indicate the reduction in anti-FXa activity compared to baseline with that volume expander. Control (green), R20/R50 = Ringer’s solution 20% or 50% dilution (purple), G20/G50 = 4% gelatine, 20% or 50% dilution (orange), 5HA20/50 = 5% human albumin 20% or 50% dilution (yellow) and 20HA20/50 = 20% human albumin 20% or 50% dilution (blue).

Median rivaroxaban concentration measured by anti-FXa activity chromogenic assay in spiked, non-diluted samples was 272 ng/mL, with an interquartile range (IQR) of 239 to 341 ng/mL. Administration of andexanet reduced the rivaroxaban concentration to a median of 7 ng/mL (IQR 1–8 ng/mL) (Figure 2).

### 3.3. Thromboelastic Coagulation Parameters

Clotting time (CT) in the control group was 86 s (IQR 67–95) at baseline. The addition of andexanet to non-anticoagulated samples prolonged CT significantly in half of the samples (control: *p* = 0.014; R20: *p* = 0.003; 5HA20: *p* = 0.003; 20HA20: *p* = 0.025). Subsequently, anticoagulation with rivaroxaban prolonged the clotting time significantly in all samples. Hemodilution further aggravated the coagulopathy, with significant prolongation observed in the G50, 5HA50, 20HA20 and 20HA50 samples. Spiking with andexanet reversed the CT in all samples close to baseline. When comparing anticoagulated samples with andexanet to non-anticoagulated samples with andexanet, a significant difference remained only in the 20% dilution with gelatine (*p* = 0.016).

**Figure 3:**
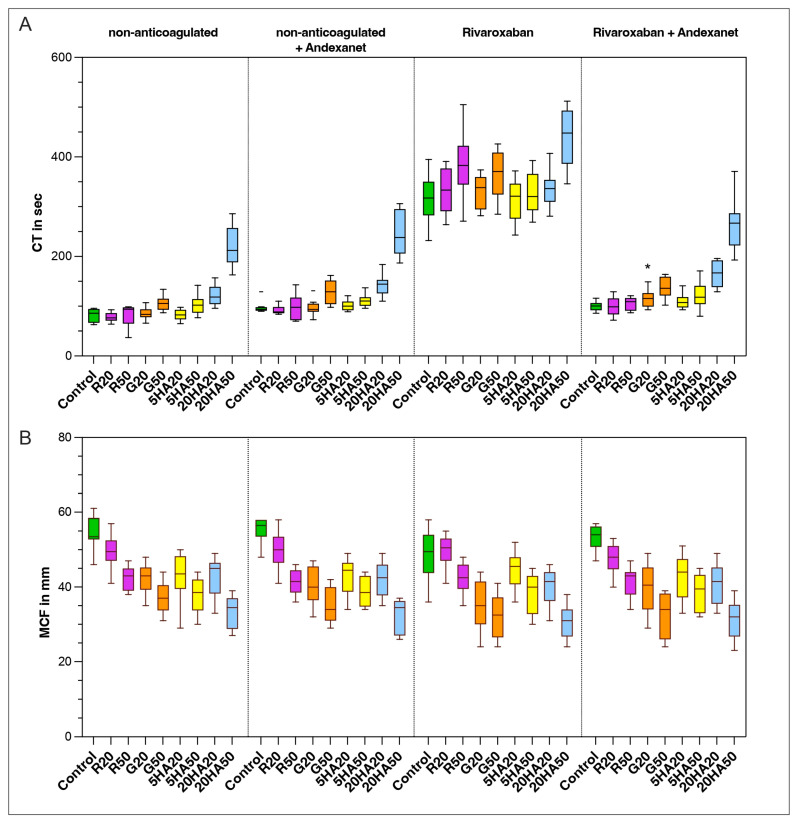
Viscoelastic parameters: (**A**) clotting time in seconds, (**B**) mean clot firmness in millimeters. Data are shown as median values, boxes show IQR and whiskers extend to the minimum and maximum values. Outliers are indicated by a line symbol. Significant differences between the “non-anticoagulated + andexanet” and the “anticoagulated + andexanet” groups are indicated by an asterisk (*). Control (green), R20/R50 = Ringer’s solution 20% or 50% dilution (purple), G20/G50 = 4% gelatine, 20% or 50% dilution (orange), 5HA20/50 = 5% human albumin 20% or 50% dilution (yellow) and 20HA20/50 = 20% human albumin 20% or 50% dilution (blue).

In the control group, mean clot firmness (MCF) was 54 mm (IQR 53–59). MCF decreased significantly upon dilution in all samples (*p* < 0.05), inversely proportional to the dilution level. Spiking of non-anticoagulated samples with andexanet had no significant effect on MCF. Anticoagulation had no significant effect on MCF (Figure 3). The only exception was the 20% dilution with gelatine (*p* = 0.017).

### 3.4. Plasma-Based Coagulation Parameters

At baseline, PT was 13 s (12–13) and aPTT was 29 s (27–30). Hemodilution prolonged PT significantly in all samples. In higher dilutions, aPTT was also significantly prolonged (G50: *p* < 0.001; 5HA50: *p* = 0.014; 20HA20: *p* < 0.001; 20HA50: *p* < 0.001). Spiking of non-anticoagulated samples with andexanet had no significant effect on PT or aPTT.

**Figure 4:**
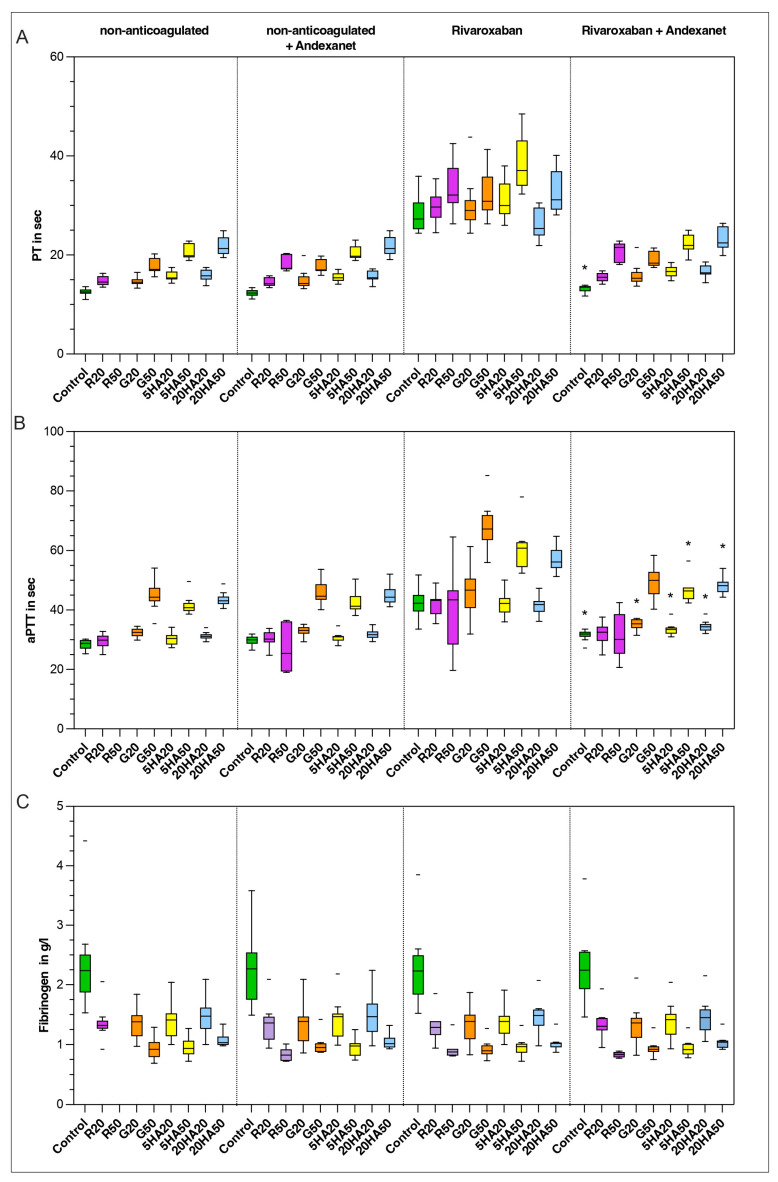
Plasmatic coagulation parameters: (**A**) prothrombin time in seconds, (**B**) activated partial thromboplastin time in seconds and (**C**) fibrinogen levels in g/L. Data are shown as median values, boxes show IQR and whiskers extend to the minimum and maximum values. Outliers are indicated by a line symbol. Significant differences between the “non-anticoagulated + andexanet” and the “anticoagulated + andexanet” groups are indicated by an asterisk (*). Control (green), R20/R50 = Ringer’s solution 20% or 50% dilution (purple), G20/G50 = 4% gelatine, 20% or 50% dilution (orange), 5HA20/50 = 5% human albumin 20% or 50% dilution (yellow) and 20HA20/50 = 20% human albumin 20% or 50% dilution (blue).

Anticoagulation with rivaroxaban significantly prolonged PT and aPTT in all samples. The addition of andexanet reversed the PT to baseline in all samples (except control: *p* = 0.017). Regarding the aPTT, there remained a significant prolongation in six of the nine samples when comparing non-anticoagulated with andexanet and anticoagulated samples with andexanet (control: *p* = 0.01; G20: *p* = 0.02; 5HA20: *p* = 0.004; 5HA50: *p* = 0.014; 20HA20: *p* = 0.003; 20HA50: *p* = 0.02). Fibrinogen levels were not altered y anticoagulation but only depended on the dilution level (Figure 4).

### 3.5. Thrombin Generation

**Figure 5:**
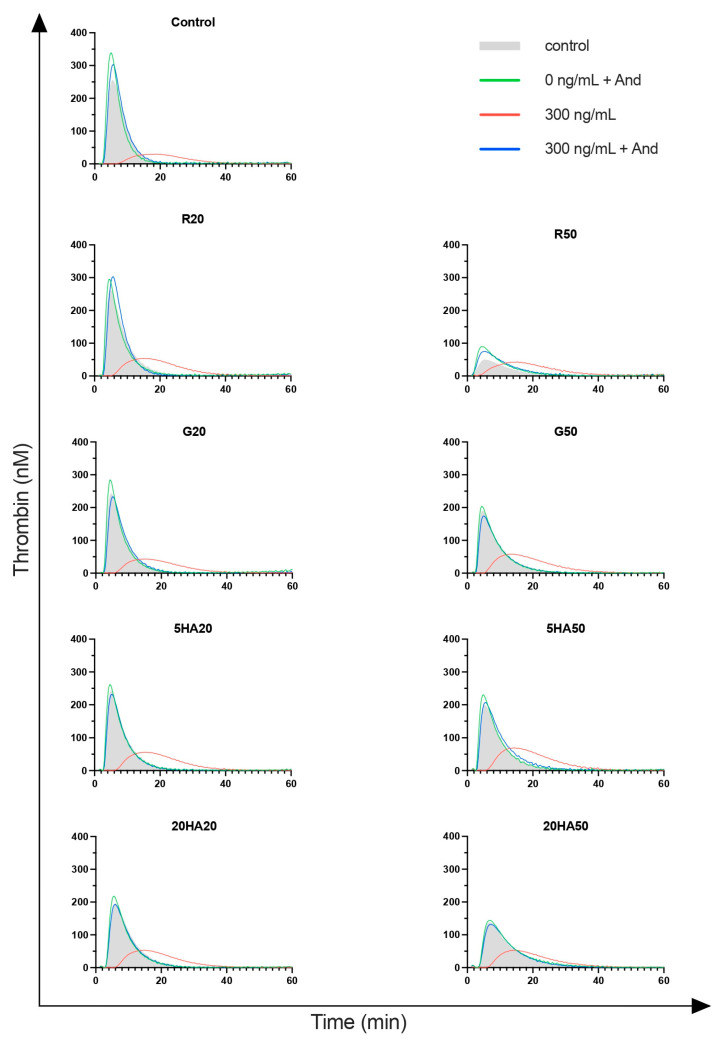
Thrombin generation profiles; n = 10 blood samples per group. Shaded area indicates anticoagulated blood.

Lagtime was initially 3.0 min (IQR 2.6–3.1) in non-hemodiluted control samples. Hemodilution had minor effects on lagtime (G20: *p* = 0.0071; 20HA20: *p* < 0.0001; 20HA50: *p* < 0.0001). Spiking of non-anticoagulated samples with andexanet did not affect lagtime. After anticoagulation, lagtime was prolonged significantly in all groups (Figure 5). The addition of andexanet reversed lagtime to baseline in nearly all groups (except G20: *p* = 0.002; 20HA50: *p* = 0.049). When comparing non-anticoagulated samples plus andexanet with anticoagulated samples plus andexanet, statistically significant differences remained in three groups (control: *p* = 0.02; G20: *p* = 0.011; 20HA20: *p* = 0.046).

Peak thrombin in control samples was 282 nM (IQR 221–343) at baseline. Hemodilution had minor effects on peak thrombin, with a significant decrease at higher dilution levels (R50: *p* < 0.001; G50: *p* = 0.015; 20HA50: *p* = 0.001). Spiking of non-anticoagulated samples with andexanet had no effect on peak thrombin. Anticoagulation significantly decreased peak thrombin in all samples except for R50. In this group, no significant effect of anticoagulation could be detected. The addition of andexanet restored peak thrombin in all samples.

Endogenous thrombin potential (ETP) was initially at 1708 nM/min (IQR 1384–1803). Hemodilution had no significant effect on ETP except for R50 (*p* < 0.001). Spiking of andexanet in non-anticoagulated samples did not affect ETP. Anticoagulation significantly reduced ETP in all samples except for R50 and 5HA50. Reversal with andexanet returned endogenous thrombin potential to baseline except for R20 (*p* = 0.029).

## 4. Discussion

In this ex vivo study, hemodilution with different volume expanders did not impair the efficacy of andexanet to reverse the anticoagulatory effects of rivaroxaban in blood samples from ten healthy donors.

Blood samples were spiked with rivaroxaban at a concentration of 272 ng/mL to mimic clinically relevant peak plasma levels [20]. Anticoagulation with rivaroxaban resulted in a significant impairment of all coagulation parameters. Hemodilution with different volume expanders further aggravated the coagulopathy.

The addition of andexanet to the anticoagulated, non-hemodiluted samples resulted in a 98% reduction in anti-FXa activity in the chromogenic anti-FXa assay. This is consistent with the findings from the ANNEXA-I and ANNEXA-4 trials [10,21], which reported a reduction in the rivaroxaban subgroups by 96% (98–93) and 94% (95–93) after andexanet administration. The anti-FXa activity in the anticoagulated, hemodiluted samples could be reduced by approximately 96% in the 20% diluted samples and by about 93% in the 50% diluted samples after the addition of andexanet. The differences between the different volume expanders at each dilution level were marginal (<1%). Our data indicate that the reversal capacity of andexanet does not depend on the choice of volume expander but only on the level of dilution. In the 50% dilution, reversal is about 5% less effective than in non-hemodiluted samples (98% reduction in control vs. 93% reduction in 50% dilution with gelatine 4%).

Viscoelastic and plasmatic coagulation parameters were reversed close to baseline levels across all samples (PT, lag time, CT, endogenous thrombin generation potential, peak thrombin) after andexanet treatment. In emergency situations, the CT of the RVV test can be especially useful as it provides a rapidly available point-of-care test (POCT) to monitor the reversal of FXa inhibitors. The aPTT could not be restored completely to baseline in all samples, but the remaining difference is unlikely to be relevant in clinical routine. Fibrinogen levels only depended on hemodilution, emphasizing the importance of close fibrinogen monitoring and substitution in major bleeding.

Hemodilution is known to impair the coagulation properties of blood by diluting important coagulation factors [22]. Fibrinogen, in particular, as the substrate of the plasmatic coagulation is of critical importance, quickly reaching low levels in bleeding patients treated with volume expanders [23]. Clotting initiation was prolonged whilst clot firmness (MCF) was reduced upon hemodilution. Visually and statistically, clot firmness depended only on dilution level. The higher the dilution, the lower the MCF. The greatest impairments of clotting parameters were observed in the 50% diluted samples, especially with 20% human albumin. Upon hemodilution, both PT and aPTT were significantly prolonged in proportion to the dilution level, whereas lag time, endogenous thrombin generation potential and peak thrombin height were only minimally affected. This may be due to the balanced dilution of pro- and anticoagulatory molecules, resulting in a maintained equilibrium of pro- and anticoagulatory effects.

In some of our whole blood samples diluted with crystalloids, the lagtime, but also ETP and peak thrombin, were significantly reduced. Potential procoagulatory effects of moderate hemodilution with crystalloids have been described previously [24]. Sinauridze et al. differentiated between crystalloids and colloids regarding their effect on coagulation when added to blood [25]. In their study, they measured an equal reduction in the concentration of procoagulants (II, X, IX, fibrinogen, V, VIII, VII, XI, XII) and anticoagulants (antithrombin III, protein C, plasminogen) after dilution, refuting approaches to explain the dilution-induced hypercoagulation by the unequal dilution of pro- and anticoagulatory factors [26]. We did not measure single factor concentrations in the plasma samples, but during the whole blood analysis, the non-anticoagulated R50 samples, in particular, tended to form clots, hampering pipetting and thrombelastic measurements. We assume that this phase led to a consumption of coagulation factors, which subsequently resulted in a reduced ETP and peak thrombin generation in these samples during TGA measurements.

We observed that the addition of andexanet to non-anticoagulated samples prolonged the initiation of clotting. The prolongation in CT may be explained by the structural similarity between andexanet and endogenous factor Xa. The competitive antagonism of these two molecules could reduce the endogenous factor Xa activity in non-anticoagulated samples [27]. Interestingly, the lag time of the thrombin generation assay could not confirm the prolongation of clotting time. A possible explanation might be the reduced levels of unbound andexanet in the plasma samples when compared to whole blood samples. However, this CT prolongation is most likely not of clinical significance.

### Limitations

Our primary focus was the assessment of pharmacodynamic parameters. To evaluate both bound and unbound andexanet levels, thus obtaining pharmacokinetic data, additional techniques such as a mass spectrometry assay could prove beneficial. Moreover, with ten donors, our sample size was rather small, not allowing for generalization of the results. Since hemodilution and andexanet administration were conducted in an ex vivo setting, further research is necessary to determine to what extent these results can be translated in vivo. In our study, dilution was conducted statically. In a clinical scenario, time points of volume resuscitation and reversal may differ. In addition, our experiment cannot account for other clinically relevant factors such as temperature, calcium levels or hypofibrinogenemia.

## 5. Conclusions

Our data suggest that the tested volume expanders do not impair the ability of andexanet to restore hemostatic capacities in rivaroxaban-spiked whole blood samples. Differences in the reduction of anti-FXa activity between the different volume expanders at each dilution level were minimal. Reversal was approximately 5% less effective at a dilution level of 50%.

## Data Availability

The data presented in this study are available on request from the corresponding author.
